# Immune evasion might affect survival in laryngeal cancer patients with blood group O

**DOI:** 10.1186/s40880-017-0223-6

**Published:** 2017-07-12

**Authors:** Kadri Altundag

**Affiliations:** MKA Breast Cancer Clinic, Tepe Prime, Cankaya, 06800 Ankara, Turkey

## To The Editor,

I want to congratulate Jin and colleagues for their article entitled “ABO blood group is a predictor of survival in patients with laryngeal cancer” recently reported in *Chinese Journal of Cancer*, in which they assessed whether the ABO blood group was associated with prognosis in patients with laryngeal cancer [1]. They reported that compared with the patients with blood group non-O, patients with blood group O had significantly shorter OS. However, there is no satisfactory explanation why laryngeal cancer patients with blood group O had shorter OS. Blood group antigens are expressed on the surface of red blood cells and numerous other tissues throughout the body, including different types of cancer cells as well [2]. These A or B antigens located on laryngeal cancer cell surface may be recognized from the immune system as antigenic foci. Hence, laryngeal cancer cells carrying A or B or both antigens could be destroyed by the immune system. On the other hand, laryngeal cancer cells harboring neither A nor B antigens, so-called type O cancer cells may not be recognized by immune cells and therefore may escape from immune destruction. Therefore, laryngeal cancer patients with blood group O might have shorter OS through the mechanism of immune evasion. This proposal has to be validated in further studies.

I confirm that I have read BioMed Central’s guidance on competing interests. I have no competing interests in the manuscript.

## The authors’ replies

We thank Dr. Kadri Altundag for the letter and the comments concerning our manuscript entitled “ABO blood group is a predictor of survival in patients with laryngeal cancer” [1]. We have studied the comments carefully, and the replies are as following.

In our study of 1260 patients with laryngeal cancer, we investigated the association between the ABO blood group and clinicopathologic characteristics and patient prognosis. We found that compared with the patients with blood group non-O, patients with blood group O had significantly shorter OS. Our findings are similar to those of previous studies on non-muscle invasive bladder urothelial carcinoma [3] and locoregional esophageal squamous cell carcinoma [4]. Our findings, however, are not in line with the findings of previous studies on pancreatic cancer [5], renal cell carcinoma [6], or curatively resected non-small cell lung cancer (NSCLC) [7]. Pancreatic cancer, renal cell carcinoma, and NSCLC patients with blood group O had a significantly longer survival than patients with blood group non-O [5–7]. Underlying mechanisms still need to be explored or confirmed.

Dr. Kadri Altundag surmised that laryngeal cancer cells harboring neither A nor B antigens, and so-called type O cancer cells may not be recognized by immune cells and therefore may escape from immune destruction; therefore, laryngeal cancer patients with blood group O might have shorter OS through the mechanism of immune evasion. It is a plausible hypothesis worthy to be tested in the further studies.

Ting Jin^1,2^, Pei-Jing Li^3^, Xiao-Zhong Chen^1,2*^, and Wei-Han Hu^3*^

^1^Key Laboratory of Radiation Oncology in Zhejiang Province, Hangzhou, Zhejiang, 310022, P. R. China

^2^Department of Radiation Oncology, Zhejiang Cancer Hospital, Hangzhou, Zhejiang, 310022, P. R. China

^3^Department of Radiation Oncology, Sun Yat-sen University Cancer Center; State Key Laboratory of Oncology in South China; Collaborative Innovation Center of Cancer Medicine, Guangzhou, Guangdong, 510060, P. R. China

*Corresponding authors: Wei-Han Hu: huwh@sysucc.org.cn; Xiao-Zhong Chen: chenxiaozhongfy@163.com

### References

1. Jin T, Li PJ, Chen XZ, Hu WH. ABO blood group is a predictor of survival in patients with laryngeal cancer. Chin J Cancer. 2016;13;35(1):90.
2. Hakomori S. Antigen structure and genetic basis of histo-blood groups A, B and O: their changes associated with human cancer. Biochim Biophys Acta. 1999;1473(1):247–66.
3. Klatte T, Xylinas E, Rieken M, Kluth LA, Roupret M, Pycha A, et al. Impact of ABO blood type on outcomes in patients with primary nonmuscle invasive bladder cancer. J Urol. 2014;191:1238–43.
4. Sun P, Chen C, Zhang F, An X, Li XY, Li YH, et al. The ABO blood group predicts survival in esophageal squamous cell carcinoma in patients who ever smoked: a retrospective study from China. Tumour Biol. 2014;35:7201–8.
5. Rahbari NN, Bork U, Hinz U, Leo A, Kirchberg J, Koch M, et al. ABO blood group and prognosis in patients with pancreatic cancer. BMC Cancer. 2012;12:319.
6. Kaffenberger SD, Morgan TM, Stratton KL, Boachie AM, Barocas DA, Chang SS, et al. ABO blood group is a predictor of survival in patients undergoing surgery for renal cell carcinoma. BJU Int. 2012;110:E641–6.
7. Li N, Xu M, Li CF, Ou W, Wang BX, Zhang SL, et al. Prognostic role of the ABO blood types in Chinese patients with curatively resected non-small cell lung cancer: a retrospective analysis of 1601 cases at a single cancer center. Chin J Cancer. 2015;34:54.

